# Description of Photographic Plates of an Edematous Acardia1From *Transactions American Obstetricians and Gynecologists*, Vol. III., 1890.

**Published:** 1891-04

**Authors:** James F. W. Ross

**Affiliations:** Toronto, Can.


### DESCRIPTION OF PHOTOGRAPHIC PLATES OF AN EDEMATOUS ACARDIA.[1]

By JAMES F. W. ROSS, M. D, Toronto, Can.

I was sent for, three months ago, to perform a Porro operation upon a patient of Drs. Britton and Shaw, of Little York. I put into my instrument valise a cranioclast and perforator, as well as midwifery forceps.

1. From *Transactions American Obstetricians and Gynecologists*, Vol. III., 1890.

On my arrival I found the patient under chloroform, with strong expulsive pains, and the two enormously-swelled legs of a fetus between her thighs. The feet were almost pulled off, showing that the doctors had been making every effort to effect delivery, but without avail. They concluded that they had a monster to deal with, and thought that it was a case of ascites. This conclusion might readily be arrived at from the peculiar formation of the child. The tissues of the leg were edematous, and I concluded, after passing my hand, that the whole monster was edematous. From this manipulation I also diagnosticated twins, and felt a large blood-clot that had been poured out from the placenta between the two children. Before proceeding to do any abdominal operation I thought it advisable to make one attempt to deliver the child from below. I therefore cut through one thigh and applied the cranioclast to the bones of the pelvis, and then by one powerful traction succeeded in effecting a spontaneous delivery of the monster shown in the accompanying plate. The other twin followed after catching a foot, and was stillborn.

*Examination:* The whole body was edematous ; the legs swelled to three or four times their natural size. The upper part consisted of a large, rounded eminence, showing on section cyst-cavities filled with a clear, straw-colored fluid, and between the cysts connective tissue with its interspaces filled with fluid. No thorax, arms, or head present. An abdominal cavity filled with intestine in a much earlier stage of development than either of the twins, and also containing two kidneys. No stomach and no liver, spleen, or pancreas to be found. The sex could not be made out. A portion of the lower end of the spinal column was present, as seen in the plate, showing a posterior view, and it contained spinal cord. There was no maceration of either child, and the other was probably alive a few hours before birth. Development had evidently been early arrested in the upper portion of the embryo. Blood was found in various parts of the fetus. The kidneys and the other parts looked pale. An interesting question arises, "How is such a fetus nourished ?" An attempt was made to inject the umbilical vessels, but was unsuccessful, owing to their brittleness. The cord looked like the cord of a fetus about the fifth month.

---

DR. E. N. BRUSH says that today no competent authority claims that diseases peculiar to women produce any considerable number of cases of insanity. *Per contra*, uterine treatment has been overdone, and gynecology has much to learn from psychiatry and neurology.—*American Lancet.*

